# Neural Substrate of Group Mental Health: Insights from Multi-Brain Reference Frame in Functional Neuroimaging

**DOI:** 10.3389/fpsyg.2017.01627

**Published:** 2017-09-28

**Authors:** Dipanjan Ray, Dipanjan Roy, Brahmdeep Sindhu, Pratap Sharan, Arpan Banerjee

**Affiliations:** ^1^Cognitive Brain Lab, National Brain Research Centre, Manesar, India; ^2^Department of Psychiatry, Gurgaon Civil Hospital, Gurgaon, India; ^3^Department of Psychiatry, All India Institute of Medical Sciences, New Delhi, India

**Keywords:** inter-subject correlation, functional neuroimaging, multi-body neuroscience, mental health, collective consciousness, interpersonal space

## Abstract

Contemporary mental health practice primarily centers around the neurobiological and psychological processes at the individual level. However, a more careful consideration of interpersonal and other group-level attributes (e.g., interpersonal relationship, mutual trust/hostility, interdependence, and cooperation) and a better grasp of their pathology can add a crucial dimension to our understanding of mental health problems. A few recent studies have delved into the interpersonal behavioral processes in the context of different psychiatric abnormalities. Neuroimaging can supplement these approaches by providing insight into the neurobiology of interpersonal functioning. Keeping this view in mind, we discuss a recently developed approach in functional neuroimaging that calls for a shift from a focus on neural information contained *within* brain space to a multi-brain framework exploring degree of similarity/dissimilarity of neural signals *between* multiple interacting brains. We hypothesize novel applications of quantitative neuroimaging markers like inter-subject correlation that might be able to evaluate the role of interpersonal attributes affecting an individual or a group. Empirical evidences of the usage of these markers in understanding the neurobiology of social interactions are provided to argue for their application in future mental health research.

## Mental Health in Individuals and Beyond

All actual life is encounter

—Buber, 1923/1970

Contemporary mental health paradigm limits mental disorders to problems within an individual. DSM-5 defines mental disorder as “a syndrome characterized by clinically significant disturbance in an individual’s cognition, emotion regulation, or behavior” (American Psychiatric Association, 2013). Accordingly, mental health interventions mostly focus on treatment of individual patients. Psychopharmacotherapy targets the neurochemical processes taking place inside individual brains. Psychotherapy usually has a focus on the psychological processes in an individual: for example, cognitive behavioral therapy focuses on an individual’s maladaptive patterns of thought and behavior, and psychodynamic psychotherapy focuses on unconscious contents of the psyche of an individual (Leichsenring and Leibing, 2007; Beck, 2011). Even interpersonal psychotherapy (IPT), though based on the recognition of the crucial role played by interpersonal factors in the genesis of mental illness, concentrates on individual sufferers in practice. During IPT sessions, a therapist guides an individual to learn appropriate emotional expressions and other skills to address problems in the relationship with significant others or to modify his/her expectation of the relationship (Markowitz and Weissman, 2004). Thus, with the exception of family and group therapies, in all major therapeutic approaches in mental health including IPT, “intra-personal” space is held as the main site of anomaly. Processes taking place in the interpersonal space are discussed as mere risk factors/predisposing factors that can causally affect the intra-personal space.

Contrary to the individual-centric approaches, an interpersonal perspective of mental health is built on the assumption that the interpersonal functioning may be deranged, *independent* of the abnormalities in individual sufferers. Thus, it aims to bring disturbed interpersonal relationships into the clinic and direct medical interventions toward the improvement of the same. Hence, interpersonal perspective necessitates a direct evaluation of the functioning of the interpersonal space comprising of the patient, related individuals, and their interactions rather than the assessment of the social and interpersonal skills of the patient as employed in approaches that focus on the individual patient. A broader clinical scope of services can be offered as impaired interpersonal spaces are present in several mental illnesses. For example, persons with paranoid personality disorder are characterized by pervasive, irrational mistrust, and suspicion of other people (Bernstein et al., 1993). The abnormality in this disorder is thus contingent on the company of other individuals and the corresponding interpersonal spaces. Likewise, an individual suffering from antisocial personality disorder has a pervasive pattern of disregard for the rights of others (Swanson et al., 1994). Dependent personality disorder is marked by an excessive psychological dependence on others (Disney, 2013). A person with social anxiety disorder has a fear of being closely watched and criticized by others (Stein and Stein, 2008). Autism spectrum disorder (ASD) is characterized by an impaired ability to communicate with other people (Lord et al., 2000). In sexual sadism disorder, sexual gratification is derived from inflicting psychological or physical sufferings on others (Hamilton and Rosen, 2016). In all these examples, pathology lies in the interpersonal space and hence a prime focus on the individual is bound to be inadequate for comprehensive understanding of the disorder in question.

Certainly, there are other disorders where abnormality can be defined within an individual or in other words in the intra-personal space. Nevertheless, all mental disorders unfold in an interpersonal context. Accordingly, interpersonal processes shape the symptomatology, therapeutic alliance, rehabilitation, and other factors that ultimately determine the course of the disease and treatment outcomes. Thus, even in treating individual patients, a direct evaluation of the interpersonal functioning may reveal important insights.

There has been a growing recognition of the importance of interpersonal functioning in mental health and the necessity to evaluate it (King-Casas and Chiu, 2012; Schilbach et al., 2013; Schilbach, 2016). A handful of recent studies have borrowed game theoretic paradigms from behavioral economics to probe interpersonal dynamics in mental health disorders. An example being the study by King-Casas et al. (2008) in which an iterated version of the economic exchange “trust” game is employed to probe the cooperation in bipolar personality disorder (BPD). Participants included normal controls paired with either another normal individual or an individual with BPD, resulting in two types of “dyads” or pairs. Compared to a normal–normal dyad, a dyad with a BPD individual showed a consistent tendency to rupture cooperation and an impaired ability to repair cooperation once it was ruptured. Another study has found greater chances of mutual defection in dyads that include high-psychopathic individuals in a cooperation game known as the “prisoner’s dilemma” (Rilling et al., 2007). Lack of facilitation of donation choices when being observed by others has also been reported employing a modified dictator game in the group comprising of autistic individuals (Yoshida et al., 2010). Neuroimaging studies have also been employed to investigate neural underpinnings of disordered social interaction. However, most of these studies, following the conventional way of reporting neuroimaging results, describe neural activity in a coordinate system constructed around a single brain, i.e., an individual brain reference frame is employed. For example, in the same BPD study described above, the researchers found decreased anterior insular cortex activation in BPD patients relative to their typically developing (TD) counterparts in the backdrop of a faltering cooperation with their partners. In spite of initial promises and more than 20 years of worldwide research efforts, neuroimaging under the individual brain-centered regime has yet to achieve the desired effect in the domain of mental health research (Linden, 2012). Although neuroimaging provides even more sensitive measures of brain structures and functions relevant for human cognitive processes, it has failed, so far, to come up with a single reliable marker for diagnosis of major mental disorders (Deacon, 2013). An attractive postulate is that much relevant information regarding mental health is contained in the interpersonal interactions that cannot be fully captured by limiting our search of markers to individual brains. This possibility calls for employing a multi-brain reference frame in functional imaging studies where interactions among multiple brains are the main object of study, rather than individual brain activations. For example, in the King-Casas et al. study, an fMRI investigation incorporating the multi-brain framework could have explored how the interdependence of the hemodynamic signals from the normal-BPD dyads differ from that of the normal–normal dyads. In the remainder of this article, we would like to draw the attention of mental health researchers, to some exciting new developments in the field of neuroimaging that hold tremendous promise to capture the interpersonal attributes of mental health. We briefly present the history of the evolution of reference frame in functional neuroimaging from single region-of-interest (ROI) to multiple brains in section “Functional Neuroimaging: Evolution of Reference Frame.” We then identify potential application areas of multi-brain neuroimaging in mental health research (section “Multi-Brain Reference Frame of Functional Neuroimaging and Mental Health Research: Some Potential Application Areas”). Following this, we further demonstrate some theoretical implications of this quantitative “description” of the interpersonal perspective of mental health and discuss why this should be seen as a compliment, *rather than a substitute*, for the existing rich body of qualitative research on this topic (section “Collective Mental State and Multi-Body Reference Frame of Functional Neuroimaging: Some Theoretical Implications”).

## Functional Neuroimaging: Evolution of Reference Frame

**FIGURE 1:**
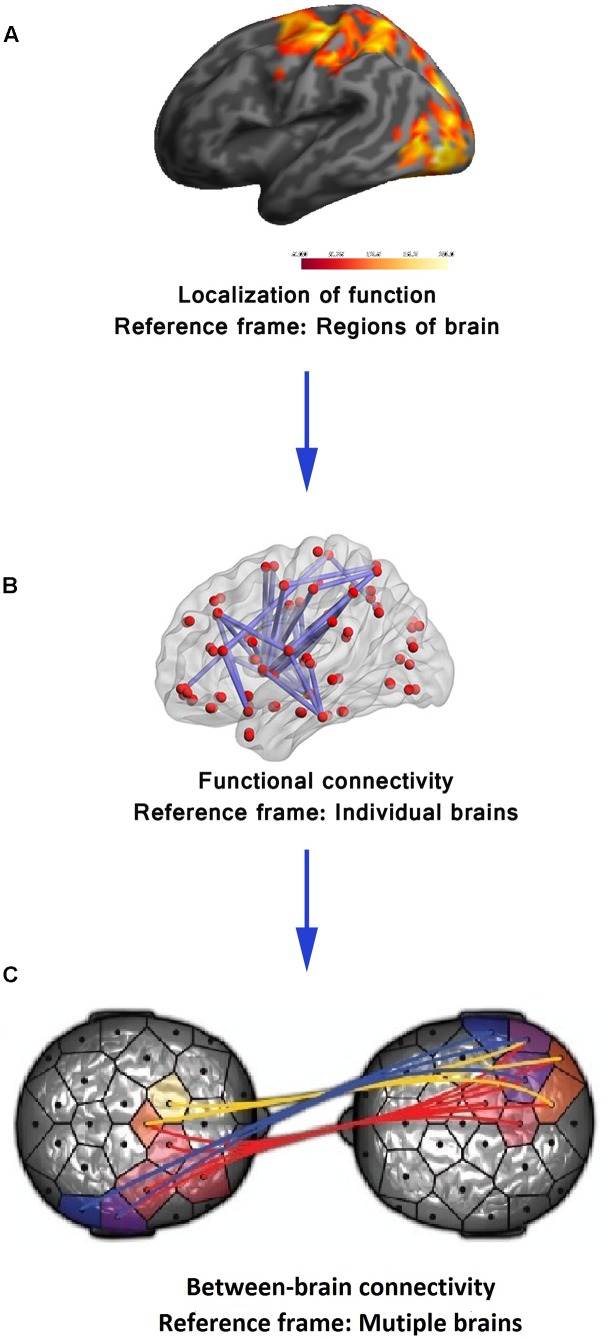
Functional neuroimaging: evolution of reference frame. **(A)** In its early years, the sole focus of functional neuroimaging techniques (e.g., fMRI) was to localize brain function to distinct brain regions. **(B)** The first paradigm shift took place with experimental validation of functional integration over distinct brain areas using measures of functional connectivity and shifting of the reference frame to the whole of the brain. Adapted from “Decreased functional brain connectivity in adolescents with internet addiction,” by Hong et al. (2013). Copyright 2013 by Hong et al. used under Creative Commons Attribution License. **(C)** The emerging techniques to assess between-brain functional connectivity calls for a second paradigm shift from a single brain to a multi-brain reference frame. Adapted from “Toward a two-body neuroscience,” by Dumas (2011). Copyright 2011 by Landes Bioscience. Adapted with permission.

The primary research strategy of functional neuroimaging in the late 20th century was the localization of a particular brain function to a distinct region of the brain (Brett et al., 2002). For example, in fMRI, the goal was to identify brain voxels showing significant relative blood oxygenation level change compared to a baseline as an indirect measure of brain activity in a particular sensory, motor, or cognitive task (Forster et al., 1998) (see **Figure 1A**). For the last two decades, however, there is a gradual shift toward a paradigm giving equal importance to functional integration of information over distinct brain areas. This shift of the reference frame from brain regions to the whole brain (see **Figure 1B**, Hong et al., 2013) is an acknowledgment of the fact that different aspects of a particular brain function are sub served in distinct brain areas but the overall performance is dependent on integration among the distinct modules across multiple spatiotemporal scales (Friston, 1994). The recognition that spatiotemporal integration of brain activity has functional implications leads to its exploration using functional connectivity analysis that measures statistical dependence in neuronal activation patterns of anatomically separated brain regions (e.g., “region A correlates with region B”) (Rogers et al., 2007). Research studies have employed effective connectivity analysis that explores how different parts of the brain impart causal influences on each other (“region A drives region B”) (Friston, 1994). The functional and effective connectivity is typically quantitated in EEG/magnetoencephalography (MEG) recordings or BOLD signals on the basis of correlation, coherence, Granger causality (GC) or transfer entropy between time series of different regions (Li et al., 2009). Several other sophisticated statistical tools including graph theoretical analysis have also been employed (Toppi et al., 2015). From the perspective of mental health, localization of functional neuroimaging, so far, has failed to identify pathology in a single brain region that is causally related to any of the major mental illnesses (Deacon, 2013). The emergence of the connectivity analysis encourages the neuroscience community to explore the possibility that more information regarding neural correlates of mental illnesses may be found at the level of the interaction of distributed neural systems rather than in discrete brain regions (Garrity et al., 2007; Greicius et al., 2007; Broyd et al., 2009; Woodward and Cascio, 2015).

**FIGURE 2:**
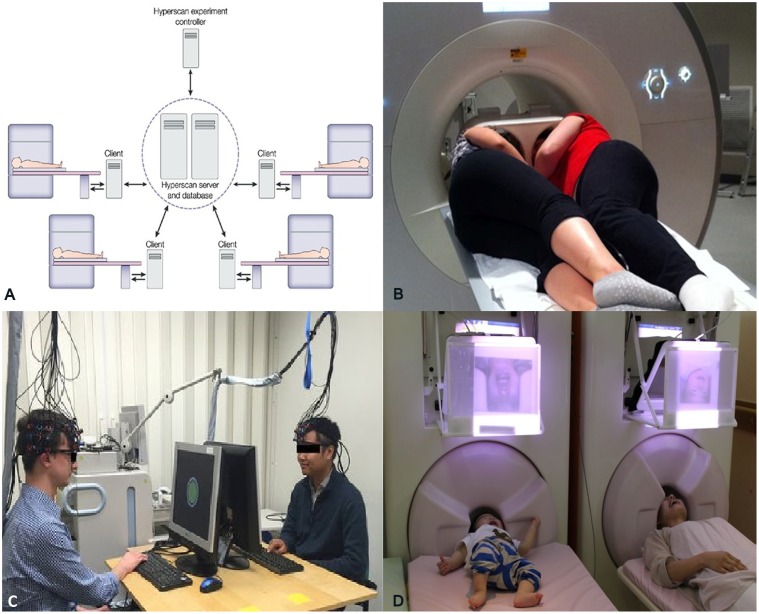
**(A)** A schematic representation of simultaneous fMRI recording using multiple scanners. Adapted from “Moral cognition and its neural constituents,” by Casebeer (2003). Copyright 2003 by Nature Publishing Group. Adapted with permission. **(B)** A specially designed dual-head coil for fMRI hyperscanning in a single scanner. Adapted from “Decoupled circular-polarized dual-head volume coil pair for studying two interacting human brains with dyadic fMRI,” by Lee et al. (2012). Copyright 2011 by Wiley Periodicals, Inc., Adapted with permission. **(C)** Simultaneous recording of Functional Near-Infrared Spectroscopy (fNIRS) data from two individuals participating in computer-based cooperation tasks using a single recording device. Adapted from “Sex differences in neural and behavioral signatures of cooperation revealed by fNIRS hyperscanning,” by Baker et al. (2016). Copyright 2016 by Macmillan Publishers Limited. Used under Creative Commons Attribution 4.0 International License. **(D)** Magnetoencephalography (MEG) hyperscanning of a mother and her infant. A mother and her infant look at each other’s facial expressions while simultaneous MEG recordings are performed in two separate scanners. Adapted from “Hyperscanning MEG for understanding mother-child cerebral interactions,” by Hirata et al. (2014). Copyright 2014 by Hirata et al. used under Creative Common Attribution (“CC BY”) version 4.0 license.

At present, the domain of functional neuroimaging may be on the brink of a second paradigm shift – quantifying the brain interactions *between* individuals transcending the boundary of the skull (Tognoli et al., 2007; Dumas, 2011; Hasson et al., 2012). In the arena of cognitive neuroscience, there is a growing recognition of the common knowledge that, in most social settings, the human brain works in interaction with other brains. In many daily cognitive tasks, from the simplest of verbal interactions between two individuals to playing games, shopping in the market, teaching in the classroom, or kissing, more than one brain cooperate/compete with each other establishing a “coupling” between themselves (Tognoli et al., 2007; Dumas, 2011; Hasson et al., 2012). The structure of this coupling shapes and constrains the activity of the individual brains to a certain extent. Therefore, a proper understanding of brain activity requires putting it in an interpersonal context. This realization is paralleled by the emergence of inter-brain connectivity analyses in functional neuroimaging (see **Figure 1C**) analogous to within-brain connectivity analysis. Various developments in neuroimaging hardware and experimental paradigm have made this shift to multi-brain reference frame possible. In fMRI studies, two subjects are typically scanned simultaneously either in two different scanners (see **Figure 2A**, Casebeer, 2003) connected over the internet (Montague et al., 2002) while they interact with each other using computer interface or in a single scanner using specially designed double-head volume coil (Lee et al., 2012) (**Figure 2B**) making face-to-face interaction possible. In EEG [and Functional Near-Infrared Spectroscopy (fNIRS)] experiments, either multiple devices are employed for recording simultaneous data from multiple subjects (Babiloni et al., 2006) or a single multichannel device is used (see **Figure 2C**, Baker et al., 2016) with a portion of the channels dedicated to each participant (Liu et al., 2016). In MEG, simultaneous recordings are usually performed in separate scanners (see **Figure 2D**, Hirata et al., 2014). However, for tasks that do not need online time-to-time interaction, e.g., studies involving social perception, simultaneous recording is not an essential requirement for studying interpersonal processing. A person’s brain activity can be recorded while she performs some socially relevant behavior (e.g., narrates a story, expresses some emotion, shows some gestures) that can be audio or videotaped and played to another person (perceiver) who is recorded in turn. This setup acts as a substitute for the live computer interface used in simultaneous “hyperscanning” paradigms described above. Thereafter, various time series analysis techniques can be used to quantify the degree of togetherness and dissimilarity between signals obtained from the two individuals.

### Inter-Subject Correlation (ISC)

**FIGURE 3:**
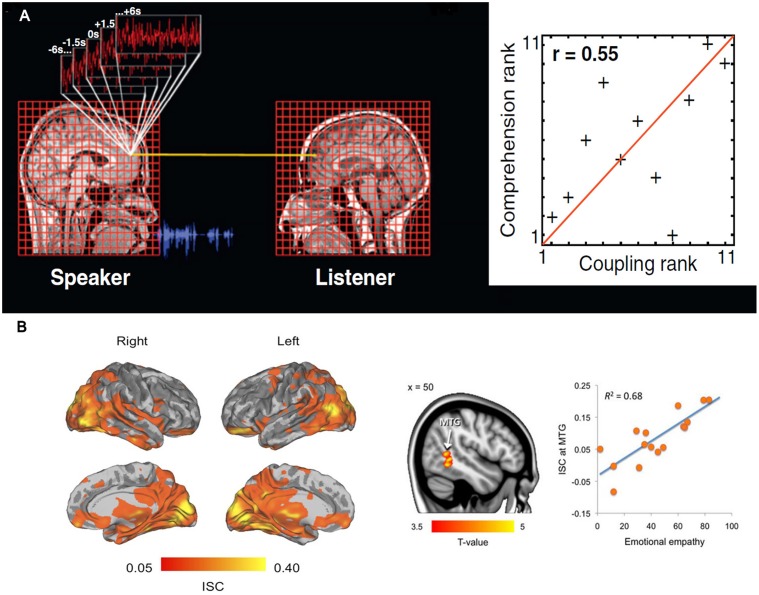
**(A)** Inter-subject correlation (ISC) between hemodynamic responses of a speaker listener pair during storytelling. The neural coupling was assessed through the use of a general linear model in which the time series in the speaker’s brain are used to predict the activity in the listeners’ brains. The second part of the figure shows that, the greater the neural coupling between a speaker and listener the better the understanding. Adapted from “Speaker-listener neural coupling underlies successful communication,” by Stephens et al. (2010). **(B)** Inter-subject correlation while watching movies depicting unpleasant, neutral, and pleasant emotions. Brain regions showing statistically significant [*P* < 0.05, false discovery rate (FDR) corrected] group-level ISCs during viewing of film clips. Tendency to catch others’ emotions as indexed by Measure of Emotional Empathy in individual subjects was associated with ISC in the right middle temporal gyrus (*P* < 0.05, FDR corrected). ISC scores in the right panel are averages from an 8-mm sphere drawn around the peak voxel. Adapted from “Emotions promote social interaction by synchronizing brain activity across individuals,” by Nummenmaa et al. (2012).

The simplest measure imaginable to quantify inter-brain neural processes is a correlation. Consequently, a multitude of studies have employed ISC analysis as a measure of information exchange between two brains. For example, in a 2010 study (Stephens et al., 2010), fMRI was recorded when a speaker narrated an unrehearsed life story within the MRI scanner. The story was recorded and later played for listeners while further fMRI scans were done. The study found strong ISC between time-locked neural dynamics of speaker’s and listener’s brain in various regions, including low-level auditory processing areas, auditory comprehension areas, sound production areas, and areas known to be involved in processing semantic and social information (**Figure 3A**). Moreover, ISC measures correlated well with the level of understanding of the story, and disappeared when there was a failure of communication. Similar neural-functional coupling has been observed during non-verbal communication involving gestures to communicate meaning (Schippers et al., 2010). Expanding the mirror neuron theory, first proposed for monkey premotor cortex (Rizzolatti et al., 1996), a more general framework suggests that understanding of the action, emotion, and sensation of other individuals crucially depends on simulation of inner state of others through activation of specific brain regions or neurons in the perceiver’s brain (Keysers and Gazzola, 2009). Thus, as the above examples show, ISC can be seen as a neural marker of successful inter-brain communications.

Understandably, among all inter-brain communications, communication of emotion is the most important process for the purpose of mental health. Several neuroimaging studies have supported the idea that emotional expression and perception are intimately related (Jackson et al., 2005; Jabbi et al., 2008). These observations have been further extended using ISC analysis. In an fMRI study, female participants were asked to freely express their emotional feeling within the scanner while their facial expressions were videotaped and shown to their romantic partners who were also being scanned (Anders et al., 2011). The subsequent ISC analysis showed significant coupling of neuronal activity between the female and male partners during sending and receiving affective cues. Recently, another study (Nummenmaa et al., 2012) found that while watching movies depicting unpleasant, neutral, and pleasant emotions, participants’ brain activity was synchronized not only in lower- and higher-order sensory areas but also in emotion processing, visual attention, and mental simulation networks (**Figure 3B**). This may reflect the neurobiological processes that help in mental simulation of other’s emotional, attentional, and sensory states by synchronizing the brain activities of specific neural circuits. This notion was further validated by the observations from the same study (Nummenmaa et al., 2012) that the ISC in the posterior middle temporal gyrus, an area that has been proposed to encode the intentions of an agent’s actions, was positively associated with self-reported empathy, i.e., the capacity to understand other people’s feelings. In other words, the higher the self-reported empathy scores were the more similar were the MTG time courses among those participants.

### Methods Beyond ISC

Correlation is a relatively crude measure of the relationship between two random variables. Consequently, other more sophisticated measures of connectivity have been borrowed from the intra-brain analysis to the inter-brain analysis. For example, several fNIRS studies (Dommer et al., 2012; Holper et al., 2012) have used wavelet transform coherence (WTC) for measuring between-brain connectivity in hyperscanning paradigms. WTC (Chang and Glover, 2010) is a method for analyzing the coherence between two time series as a function of both time and frequency. WTC is capable of detecting significant coherence between two time series even in the face of low common power. Similarly, researchers (Yun et al., 2012) have employed phase locking value (PLV), a measure of consistency in the relationship of the phases of two EEG signals with respect to time (Lachaux et al., 1999), as a marker of social interaction. In contrast to analyses based on frequency specific spectral changes, phase synchronization is said to be particularly suitable for quantifying long-range functional connectivity which may be critical for understanding neural mechanisms during interpersonal interactions. In many social situations, the directed asymmetric influence of one individual to another individual, rather than simple connectivity estimates, might be of particular interest and hence causality analysis may provide further useful information. Accordingly, both GC (Holper et al., 2012) and frequency domain variances of GC like partial directed coherence (Dumas et al., 2010; Astolfi et al., 2011) have been undertaken in inter-brain analyses. Additionally, as it is well established that several functional aspects of a complex system like the brain show strong non-linearities across levels of organizations, some of the tools of non-linear dynamics have been proposed to investigate the neural dynamics of inter-brain interactions (Tognoli and Kelso, 2009). In particular, transfer entropy, a model free information theoretic measure of effective connectivity, has been suggested (Liu and Pelowski, 2014) to be well-suited for this purpose. The graph theory approach has also been extended to describe the functional topology formed by multiple interacting brains (Toppi et al., 2015).

## Multi-Brain Reference Frame of Functional Neuroimaging and Mental Health Research: Some Potential Application Areas

We propose that the development of inter-brain connectivity analyses may have important implications for mental health research. Recognizing other individual’s affective, sensory, and attentional states provides the key to predicting their intentions and actions. Emotional intelligence (EI), sometimes described as the ability to identify and manage our own emotions and the emotions of others (Goleman, 2006), has become of widespread interest to psychological research in recent years (Hertel et al., 2009). Both EI and empathy have been reported to be associated with mental health status both at the individual (Hertel et al., 2009; Castro, 2011) and organizational (Slaski and Cartwright, 2003) levels. Measures of inter-brain neural processing can be used as objective markers for mutual empathy and interpersonal understanding between individuals. Though there are several commentaries on inter-brain connectivity analyses, their implications in mental health research are yet to be properly discussed. In what follows, we identify certain research areas in mental health discourse that include both the mental health conditions and other related issues like rehabilitations and therapeutic alliance where the interpersonal perspective is prominent and hence inter-brain connectivity analyses may find some applications. To the best of our knowledge, this is the first paper to offer a framework for the application of this novel approach in mental health research. Understandably, at present, most of the hypothetical applications proposed below lack any empirical validity. Still, they sub serve two main purposes here: they provide real life hypothetical examples for a better understanding of the concepts presented in this paper; and they also propose a roadmap for a meaningful future collaboration between mental health and neuroimaging researchers to explore this state-of-the-art methodology.

### Mental Health Disorders

#### Depression

A rich body of literature exists discussing the interpersonal perspective in depression and other mood disorders. Several studies have identified, for example, basic behavioral differences between depressed and non-depressed individuals in interpersonal contexts (Hames et al., 2013). Depressed individuals have been found to be less likely to initiate social interactions (Libet and Lewinsohn, 1973), to speak more slowly with less volume and voice modulation (Youngren and Lewinsohn, 1980), engage in less eye contact (Kazdin et al., 1985), hold their head downward and engage in more self-touching (Ranelli and Miller, 1981), and use fewer gestures (Kazdin et al., 1985) compared to non-depressed individuals while communicating with others. Thus, hyperscanning depression patients and their friends/relatives during social interactions can be a useful tool to identify neural markers of impaired interpersonal communication in depression. Particularly, it can be tested as a tool to monitor progress in patients under interpersonal therapy. In addition, inter-brain connectivity analysis can also be helpful in the case of depression contagion. There is empirically validated evidence that shows close contacts, such as roommates of patients suffering from depression have higher chance to get affected, even after controlling for the shared negative life stress (Haeffel et al., 2014). Most interestingly, in the context of mental health, studies have empirically verified that caregiver’s emotional empathy is positively correlated with their mental health outcomes (Lee et al., 2001; Ruiz-Robledillo et al., 2015). Inter-brain connectivity, as a neuroimaging marker of mutual empathy, has the potential to become a key tool in the research on depression contagion.

#### Autism Spectrum Disorders (ASD)

A core feature of ASD is impairment of reciprocal social interactions (Kanner, 1968). Therefore, it is not surprising that ASD is perhaps the only mental disorder where ISC analysis has already been applied. An fMRI study using ISC analysis revealed that activity in the right inferior frontal gyrus (IFG) is diminished in TD–ASD pairs compared to TD–TD pairs during joint attention task (Tanabe et al., 2012). Another fMRI study showed that during watching a movie portraying social interaction, ASD participants show diminished ISC in brain regions implicated in processing social information, including the insula, posterior and anterior cingulate cortex (PCC/ACC), caudate nucleus, precuneus lateral occipital cortex, and supramarginal gyrus (Salmi et al., 2013). Both studies employed only adult ASD patients. Nonetheless, inter-brain connectivity approach can also be particularly useful in child ASD subjects as several studies have stressed the need for early diagnosis of ASD. At present, ASD is usually detected at 3–6 years of age (Landa, 2008) and can be diagnosed as early as the age of two (Charman and Baird, 2002). Several existing studies have suggested that an detection and intervention notably improves prognosis (Nadel and Poss, 2007; Dasgupta et al., 2016). Inter-brain connectivity measures can be employed to explore social interaction between children and their caregivers for this purpose. Recent development of a hyperscanning system to examine real-time brain-to-brain interaction between a mother and her child (**Figure 2D**) may prove useful in this regard (Hirata et al., 2014).

#### Schizophrenia

According to a theory first proposed in 1992 (Frith, 1992), a wide range of symptoms in schizophrenia can be explained in terms of compromise in the theory of mind (ToM). For example, inability to attribute mental state to own behavior has been postulated to be behind delusion of alien control, command hallucination, and other “passivity” symptoms (Brüne, 2006). Similarly, an abnormal mentalizing of other person’s thoughts and intentions has been suggested to be associated with symptoms like delusions of reference and persecution (for an alternative theory of impairment of ToM in schizophrenia, see Hardy-Bayle et al., 1994). For example, Abu-Akel and Bailey (2000) suggest that in some schizophrenic patients with positive symptoms, hyper-ToM with over-attribution of intentions to themselves and others may be the main psychopathology. Consequently, several studies have used different ToM tasks in schizophrenic subjects to explore these theories. Inter-brain connectivity measures can further add to this line of research by providing a neuroimaging marker of “theory of other mind” (ToOM) deficit in schizophrenia. Brain to brain connectivity can also play important part in research aiming at early diagnosis of schizophrenia as several studies have suggested that impaired interpersonal skill in childhood and adolescence is an early predictor of the development of schizophrenia in adult life (Dworkin et al., 1993; Stanghellini, 2004).

#### Personality Disorders

As stated by Sullivan (1953), personality is “the relative enduring pattern of recurrent interpersonal situations which characterize a human life.” Naturally, interpersonal factors have been suggested to form the core of the personality psychopathology (Hopwood et al., 2013). In addition to the three personality disorders mentioned in the beginning, interpersonal perspective is crucial for conceptualizing many other personality disorders. Borderline personality disorder is characterized by a pervasive pattern of instability in interpersonal relationships (Leichsenring and Leibing, 2007). Histrionic personality disorder is marked by exaggerated attention-seeking behavior (Weiner and Craighead, 2010). Narcissistic personality disorder is characterized by pervasive grandiosity, an excessive need for admiration and a lack of empathy for others (Ritter et al., 2011). Persons with avoidant personality disorder persistently show social inhibition, feelings of inferiority, and extreme sensitivity to negative criticism or rejection by others (Weiner and Craighead, 2010). Thus, it will be interesting to investigate any differences in inter-brain connectivity patterns in the context of different personality disorders. Additionally, a study (Alden and Capreol, 1993) on avoidant personality disorder has found that treatment response is significantly influenced by differences in patterns of interpersonal behavior among patients. Thus, between-brain analysis can be tested as a tool for treatment choice in personality disorders.

#### Social Anxiety Disorder

Interpersonal context is prominent in social anxiety disorder. Positive qualities in friendship or romantic relationship are interpersonal factors that can protect against social anxiety whereas relational victimization or negative interactions can predict high social anxiety (La Greca and Harrison, 2005). More interestingly, studies have demonstrated that high socially anxious persons show elevated mentalizing and empathic abilities. There is some suggestion that a unique social-cognitive ability profile is behind their preoccupation with the other person’s impression of them (Tibi-Elhanany and Shamay-Tsoory, 2011). Understandably, inter-brain connectivity analyses can be applied to further explore this hypothesis.

#### Somatic Symptom Disorder

Somatic symptom disorder (SSD) is characterized by excessive thoughts, feelings, and behaviors that focus on physical symptoms such as pain or fatigue. These symptoms may or may not be related to any physical cause (Kanaan et al., 2010; Rowe, 2010). Despite tremendous theoretical and research efforts, proper understanding of the psychopathology of this disorder remains elusive (Rofé and Rofé, 2013). There are some suggestions that interpersonal factors play a significant role in it. The somatizing patient often seeks the “sick” role, which provides them relief from stressful interpersonal expectations as a “primary gain,” often accompanied by attention, caring, and sometimes even monetary reward (“secondary gain”) (Moran et al., 2010). Consistent with this view, others have found desire for interpersonal closeness combined with the fear of being rejected is the most prominent internal representation of relationships in majority of patients with SSD (Landa et al., 2012). Accordingly, an interpersonal neuroimaging marker can be envisaged as a useful research tool for this mysterious disorder.

#### Eating Disorders

Eating disorders are among the list of conditions, besides depression, where the application of interpersonal therapy has been extended (Rieger et al., 2010). This is based on the recognition that interpersonal factors play the central role in the development and maintenance of eating disorder such as anorexia, bulimia, and binge eating disorder. The core behavioral patterns of eating disorders, i.e., distorted body image, preoccupation with control of body shape and weight, etc., are intimately associated with social settings in which these disorders take place, e.g., the “cult of thinness” and body shaming of contemporary popular culture (Hesse-Biber, 1996; Hesse-Biber et al., 2006). In parallel, studies have revealed significant interpersonal attributes, non-assertivity, submissive interpersonal styles, and social inhibition in patients with eating disorders (Hartmann et al., 2010). Thus, probing the neural coupling between the patient and persons in their immediate surroundings can be proved useful as a research tool in eating disorders.

#### Sexual Dysfunctions

In recent years, there is an increased research interest in various sexual dysfunctions including erectile dysfunction, arousal disorders, and sexual pain disorders. In spite of the moderate efficacy rates of the available treatment options, the discontinuation rates of treatments are equally impressive (Althof, 2002). Among the factors responsible for discontinuation, relationship issues occupy an important place. They are so intimately related to sexual functioning that in most cases it is impossible to determine which came first, a non-intimate relationship leading to sexual dysfunction or sexual dissatisfaction leading to mutual antipathy (McCabe et al., 2010). Despite this difficulty to ascribe causality, literature clearly suggests that addressing issues with interpersonal relationship is associated with better long-term outcome (Althof, 2002). This is also the premise of application of interpersonal therapy in sexual dysfunctions. Inter-brain connectivity analyses have multiple potential research applications here: from identifying the interpersonal problems behind sexual dysfunctions to shaping an outcome measure of treatment including monitoring interpersonal therapy.

#### Suicide

The presence of a mental disorder including mood disorder, schizophrenia, personality disorder, and substance abuse is an important risk factor for suicide. According to some estimate, diagnosable mental illness is present in 98% of those who commit suicide (Bertolote and Fleischmann, 2002). Assessment of suicidal risk in turn determines treatment choice and urgency of intervention in mental illnesses. Interpersonal theory of suicide (Joiner, 2007) has recently proposed an empirically validated theoretical framework that explains suicidal behavior and guide risk assessment and intervention in clinical settings. Thwarted belongingness and perceived burdensomeness are two interpersonal risk factors for suicide, as identified by this theory. Inter-brain connectivity analyses can be employed to explore the interpersonal factors between mentally ill patients and family members/friends to identify persons at risk of suicide and guide treatment strategy.

### Other Issues Related to Mental Health

#### Therapeutic Alliance

Therapeutic alliance, broadly defined as collaborative bond between patient and therapist (Krupnick et al., 1996), is considered as “the quintessential integrative variable” (Wolfe and Goldfried, 1988) of psychotherapies as mounting evidence suggests that it is a better predictor of outcome across a wide range of psychotherapeutic modalities and many other sophisticated factors (Horvath and Symonds, 1991; Martin et al., 2000; Castonguay et al., 2006). Remarkably, a few studies have provided evidence supporting the significant impact of quality of therapeutic alliance on the clinical outcome of pharmacotherapy as well (Krupnick et al., 1996; Zilcha-Mano et al., 2015). These findings stress the need for early detection and repair of “rupture” of the therapeutic alliance. Here, inter-brain connectivity between the patient and the therapist can be evaluated as a key measure of deterioration in the relationship. An objective assessment of the alliance is particularly important keeping in mind the evidence showing a marked difference in the perception of rupture between the patient and the therapist (Safran et al., 2002).

#### Rehabilitation

The goal of psychiatric rehabilitation is to help persons suffering from severe mental illnesses to assimilate in the social life seamlessly with the least amount of professional support (Rössler, 2006). The role of rehabilitation in mental health has become particularly important after the de-institutionalization movement. Interpersonal aspects play an important role in rehabilitation and this provides scope for the brain-to-brain neuroimaging. It may be investigated as a tool to assess the interpersonal skill of the patient to guide the selection of rehabilitation strategy (Goldsmith and McFall, 1975; Halford and Hayes, 1991). Moreover, inter-brain connectivity between the patient and near ones can be explored as a marker of social support as a part of resource assessment (Buchanan, 1995).

## Collective Mental State and Multi-Body Reference Frame of Functional Neuroimaging: Some Theoretical Implications

The truth is the whole.

—Hegel, 1807/1977

Throughout history theorists from disparate disciplines have engaged with the concept of groups as the bearer of mental states. In the modern era, Durkheim (1893) formulated his concept of “conscience collective” (translated as collective consciousness or collective conscience) as a set of shared beliefs, ideas, and moral attitudes that operate as a unifying force within the society. Durkheim contended that the concept of “*collective consciousness*” must be designated by a special term, simply because the states which constitute it differ specifically from those which constitute the individual consciousnesses. Based on the same concept, Durkheim methodically explored, perhaps for the first time, the social rather than individual causes of suicide including lack of social integration and solidarity, mismatch of the individual and collective moral values, excessive social control, and so on (Durkheim, 1897). Le Bon (1896) proposed that in a crowd the individual psychology is submerged in the collective mentality that completely transforms individual behavior. List of related early theoretical approaches that deal with the mental states beyond individual level includes “group psychology” by Freud (1922), “collective unconscious” by Jung (2014), “collective madness” by Borkenau (1981), etc. In spite of these early interests, the concept of collective mental state, until recently, has fallen into neglect for several reasons. One major obstacle has been the lack of direct objective measures of “collective consciousness.” As one commentator notes, “the obvious methodological problem of how such an entity could be tested empirically has been such as to place it outside modern social science discussion, which is predominantly quantitative” (Bostock, 2002, p. 2).

Development of neuroscience more firmly established the correlation of mental states with the brain processes. As a group does not possess a brain and as no neurological correlates of group mental state could be envisaged, the usage of terms like group mental state or collective mental illness became a mere metaphor with no real resemblance with the “actual” mental states or mental illnesses “taking place within a brain.” As a natural consequence, in scientific discourse, for a considerable period of time, the idea of collective consciousness had more metaphysical and transcendental connotation.

From what we have discussed so far in the present article, there are ample reasons to believe that inter-subject brain connectivity analysis (see “Glossary”) can provide some novel insights into these debates. By shifting the functional neuroimaging to the multi-brain reference frame, it can furnish an objective measure of the collective mental state, thus making it more empirically accessible. The group mental state can be investigated recruiting the same analytical tools (e.g., correlation, coherence of neural dynamics) that are employed in individual brain. By doing so, inter-brain connectivity analysis creates some sort of neural correlates of group mental state, thus giving it a firm grounding in material reality. More generally, by providing support for a neural substrate of the collective mental state our proposal contributes to the endeavor to discard dualism in the context of mental health (Kelso and Engstrøm, 2006). The repudiation of dualism can only be seen as a part of the search for a more comprehensive approach to mental health. An objective neuromarker of collective mental state can never belittle the importance of a thorough subjective assessment of psychiatric patients, nor can it ever provide the exclusive information that is contained in the individual or ROI level neuroimaging. On the contrary, by putting the individual in the interindividual context and by providing a quantitative correlate of subjective experience, it can add new dimensions to both individual-centered and qualitative research programs. We fully agree with Lewontin et al. (1984, p. 282) when they commented that

The biological and the social are neither separable, nor antithetical, nor alternatives, but complementary. All causes of behavior of organisms, in the temporal sense to which we should restrict the term cause, are simultaneously both social and biological, as they are available to analysis at many levels. All human phenomena are simultaneously social and biological, just they are simultaneously chemical and physical. Holistic and reductionist accounts of phenomena are not “causes” of those phenomena but merely “descriptions” of them at particular levels, in particular scientific languages. The language to be used at any time is contingent on the purpose of the description.

What we propose in the present article thus should be conceptualized as a novel addition to the rich array of “descriptions” of mental health phenomena. In the context of mental health, the insights that inter-brain connectivity analyses bring should be properly contextualized within the social, biological, and psychological determinants of mental health, as a complementary tool to other methods. Our ultimate goal should be to open up the discussion to a plurality of explanatory principles.

### Glossary

#### Blood Oxygen Level Dependent (BOLD) Signal

An indirect measure of neuronal activity employed in fMRI studies that relies on regional differences in cerebral blood flow and oxy-hemoglobin level.

#### Correlation

A statistical measure that quantifies the strength of interaction between two random variables (e.g., neural signals from two different areas).

#### Coherence (Signal Processing)

Frequency domain description of correlation (above).

#### Functional Near-Infrared Spectroscopy (fNIRS)

A non-invasive, low-cost functional neuroimaging tool to monitor brain activation in the pre-frontal cortex by measuring changes in near-infrared light.

#### Functional Integration

The process by which segregated brain regions at various levels work together to process information and effect responses.

#### Granger Causality

A statistical measure of directionality between two random variables. According to GC, if a signal *X* “Granger-causes” a signal *Y*, then past values of *X* should contain information that helps predict *Y* above and beyond the information contained in past values of *Y* alone.

#### Graph Theory

A mathematical formalism concerned about how networks can be symbolically represented and accounted statistically.

#### Hyperscanning

A functional neuroimaging method for measuring brain activity simultaneously from two (or more) subjects. This can become a key tool in the context of studying group mental health and needs to be explored by future investigations. Please also refer to Montague et al. (2002) for details.

#### Inter-Subject Brain Connectivity

A key measure that can be defined by computing correlation, coherence, and other statistical properties between brain signals coming from two different brains. This is a fundamental marker for the multi-brain reference frame hypothesis and can become a clinical marker.

#### Localism (Neuroscience)

A philosophical approach to neuroscience that emphasizes the functional specificity of individual brain regions.

#### Magnetoencephalography (MEG)

A non-invasive neurophysiological technique for recording the magnetic fields generated by neuronal populations at macroscopic scale.

#### Partial Directed Coherence

A frequency domain description of GC between multivariate time series represented by autoregressive model.

#### Phase Locking Value

A measure of consistency in the relationship of the phases of two signals with respect to time.

#### Transfer Entropy

An information-theoretic measure of directionality between two random variables.

#### Wavelet Transform Coherence

Coherence as a function of both time and frequency.

## Author Contributions

DRa and AB conceived the original idea. DRa wrote the manuscript with support from AB, DRo, PS, and BS. All authors provided critical feedback. AB and DRo helped supervise the project.

## Conflict of Interest Statement

The authors declare that the research was conducted in the absence of any commercial or financial relationships that could be construed as a potential conflict of interest.
